# Comparison of two disease‑specific instruments assessing health-related quality of life in patients with chronic otitis media

**DOI:** 10.1007/s00405-021-06702-y

**Published:** 2021-03-31

**Authors:** Robert Mlynski, David Bächinger, Theresa Langanke, Susen Lailach, Marcus Neudert, Nora M. Weiss

**Affiliations:** 1 Department of Otorhinolaryngology, Head and Neck Surgery, “Otto Körner”, Rostock University Medical Center, Doberaner Strasse 137-139, 18057 Rostock, Germany; 2 Department of Otorhinolaryngology, Head and Neck Surgery, University Hospital Zurich, Zurich, Switzerland; 3 University of Zurich, Zurich, Switzerland; 4 Department of Otorhinolaryngology, Head and Neck Surgery, “Carl Gustav Carus”, Dresden University Medical Center, Dresden, Germany

**Keywords:** Cholesteatoma, Questionnaire, COMOT-15, ZCMEI-21, Gender difference, Hearing perception, Concurrent validity

## Abstract

**Purpose:**

Evaluating the current health state in chronic otitis media (COM), audiologic results are complemented by subjective outcomes, such as health-related quality of life (HRQoL). Two disease-specific instruments assessing HRQoL in COM in German-speaking patients exist, i.e., the chronic otitis media outcome test (COMOT-15) and the Zurich chronic middle ear inventory (ZCMEI-21). Since the psychometric properties of these questionnaires in a concurrent application are unknown, the aim of this study was to compare the COMOT-15 and the ZCMEI-21.

**Methods:**

HRQoL was assessed in adult COM patients using the COMOT-15 and the ZCMEI-21. Psychometric properties were determined, including response distribution, concurrent validity, internal consistency, correlation to hearing and gender differences.

**Results:**

In 173 patients (mean age 51.5 years), both questionnaires showed normally distributed scores without strong floor and ceiling effects. The total scores and subscores of both questionnaires exhibited satisfactory internal consistency (Cronbach’s α 0.7–0.9) with the exception of the COMOT-15 hearing subscore (α = 0.94) and the ZCMEI-21 medical resource subscore (α = 0.66). Fair correlations between the air conduction pure-tone average and the total scores were found (COMOT-15: *r* = 0.36, *p* < 0.0001; ZCMEI-21: *r* = 0.34, *p* < 0.0001).

**Conclusion:**

In the first study comparing the COMOT-15 and the ZCMEI-21, both questionnaires exhibited satisfactory psychometric properties with several subtle differences. The COMOT-15 has a strong focus on hearing with a probably redundant content of the hearing subscore and may be suited for hearing-focused research questions. The ZCMEI-21 provides a comprehensive assessment of the COM symptom complex and may therefore also be used in research settings, where ear discharge, vertigo or pain should be covered.

## Introduction

The most frequent causes of conductive hearing loss are chronic inflammatory diseases of the middle ear, such as chronic otitis media (COM) with persistent discharge due to a chronic tympanic membrane perforation with or without cholesteatoma. Without adequate treatment, COM symptoms such as hearing loss or ear discharge can severely impair health-related quality of life (HRQoL) [1–3]. When evaluating current health state and outcomes of surgical therapies, in particular when novel surgical techniques or new prostheses are introduced, standardized reporting methods for procedures and outcomes are necessary for an objective and meaningful analysis. Since data collection in clinical otologic studies on COM not always follows uniform and systemic rules, the evaluation of surgical therapies and a comparison between different clinics and surgical schools may be difficult [4, 5]. In an attempt to achieve uniform reporting, several disease classification systems have been proposed in the past and guidelines for reporting hearing have been established [4, 6–12]. Further, the assessment of subjective outcome parameters, such as HRQoL, has gained importance to describe current health state as an indicator of therapy success in the treatment of COM. The assessment of HRQoL has become increasingly important in both, clinical trials and clinical practice [13–17]. COM may severely impair HRQoL by hearing impairment [3, 18, 19], but also by ear discharge, otalgia or dizziness [3, 15, 20].

Validated and standardized questionnaires are used to assess the psychosocial impairment caused by hearing disabilities and accompanying symptoms such as tinnitus [21, 22]. Regarding COM, two disease-specific questionnaires have been developed for adult German-speaking COM patients, i.e. the Chronic Otitis Media Outcome Test (COMOT-15) and the Zurich Chronic Middle Ear Inventory (ZCMEI-21) [23, 24]. Both the COMOT-15 and the ZCMEI-21 assess symptoms of COM and their influence on HRQoL [23–27]. Both questionnaires have subscores dedicated to ear symptoms, hearing-related problems, psychosocial impairment of COM as well as the use of medical resources. Although both questionnaires are increasingly used in research and clinical practice [15–17, 20, 28], information on the psychometric properties of the individual questionnaires in a concurrent application is not available. Yet, these data are crucial when deciding which instrument should be used for clinical practice or research studies. In this study, the aim was therefore to evaluate and compare two disease-specific questionnaires for the assessment of HRQoL.

## Materials and methods

### Study design and patient selection

Adult patients with COM before surgical intervention were recruited from two tertiary hospitals (University Medical Center, Rostock, Germany; University Medical Center, Dresden, Germany). The study protocols were approved by the local Ethics Committees in accordance with the Helsinki declaration (Registration number: A2017-0101 [Rostock], EK 166042017 [Dresden]). Informed consent was obtained from all the participants.

### Audiometric assessment

All audiometric measurements were performed with calibrated instruments in a sound-proof room (DIN EN ISO 8253). Measurements included standard pure-tone audiometry, performed with a clinical audiometer (AT1000, Auritec, Hamburg, Germany) in 5 dB steps. Pure-tone average (PTA) of the air conduction (AC) was calculated from AC thresholds at 0.5, 1, 2, and 3 kHz (PTA_0.5–3 kHz_) according to the Committee on Hearing and Equilibrium guidelines [11]. The Air bone gab (ABG) was calculated as the difference between the PTA_0.5–3 kHz_ of the bone conduction (BC) threshold and the AC threshold.

### Assessment of HRQoL

HRQoL was assessed by both the ZCMEI-21 and the COMOT-15 applied at the same time. The COMOT-15 is a disease-specific instrument assessing HRQoL in patients with COM and was developed in 2009. It consists of one total score including three subscores that cover ear-related symptoms, hearing, psychosocial effects and two additional questions addressing the number of consultations of an otolaryngologist and an overall estimation of the HRQoL. The COMOT-15 is presented using a six-point Likert scale and scored as 0–5. The individual scores are normalized to values between 0 and 100 by dividing the sum of the score by the sum of the score range and then multiplying with 100. Higher scores in the COMOT-15 overall score correlate with a poorer quality of life.

The ZCMEI-21 was developed as a disease-specific questionnaire for assessing the HRQoL in patients with COM. The ZCMEI-21 has been translated into several languages [26, 27, 29, 30] and has been successfully used in clinical studies [15, 17, 25]. The ZCMEI-21 consists of four subscores that cover ear-related symptoms, hearing, psychosocial effects of the disease and the use of medical resources. The answers are presented using a five-point Likert scale and scored as 0–4. The maximal ZCMEI-21 total score is 84. Higher scores in the ZCMEI-21 overall score correlate with a poorer quality of life. The ZCMEI-21 is the only disease-specific instrument for COM, in which the minimal clinically important difference has been determined and estimated to 5 [16].

The questionnaires were applied at an active stage of the disease, i.e. either during the outpatient visit when the diagnosis was established and surgery was indicated, or preoperatively during the hospital stay for surgery. Thus, the questionnaires were completed in the waiting room or on the ward. All patients received an instruction on how to complete the questionnaire and had the opportunity to ask questions on the questionnaire, however, no active assistance was provided.

### Statistical analysis

All statistical tests were selected before data collection. Statistical analyses were performed using Microsoft Excel (version 15.29, Microsoft Corporation, Redmond, WA, USA) and Prism (version 8, GraphPad Software, La Jolla, CA, USA). The significance level was set to *p* < 0.05. The assumption of normality was tested graphically using quantile–quantile plots. If not otherwise specified, data are presented either as mean with standard deviation (SD) or 95% confidence interval (95% CI) or as absolute numbers with percentages.

Response distribution was assessed by determining the floor and ceiling effect, i.e. the percentage of patients exhibiting the lowest or highest possible score. Further, the skewness *γ* and kurtosis *κ* were assessed. A normal (Gaussian) distribution is characterized by a skewness of 0 and a kurtosis of 0.

Internal consistency as an indicator of reliability was assessed using Cronbach’s α [31]. An α ≥ 0.7 indicates satisfactory internal consistency [31] and values > 0.90 most likely indicate unnecessary redundancy [32]. Convergent validity between the COMOT-15 and the ZCMEI-21 was assessed by calculating Spearman’s correlation. Further, correlation between hearing and the questionnaire scores was determined by calculating Spearman’s correlation. Gender differences within the individual questionnaire total scores and subscores were assessed using the Mann–Whitney *U* test.

## Results

A total of 173 patients with a mean age of 51.5 years completed both the COMOT-15 and the ZCMEI-21. Detailed demographics and patient characteristics are given in Table 1.

**Table 1:** Demographics, clinical characteristics as well as mean COMOT-15 and ZCMEI-21 scores of the study cohort

	Patients (*n* = 173)
Mean age—years (SD)	51.5 (SD 16.3)
Sex, female:male—*n* (%)	87 (50.3):86 (49.7)
COM type—*n* (%)	
COM without cholesteatoma	97 (56.1)
COM with cholesteatoma	76 (43.9)
COMOT-15 score—points (SD)	
Total score	40.8 (19.5)
Ear symptoms	30.3 (19.3)
Hearing	60.0 (26.3)
Psychosocial impact	42.2 (26.6)
HRQoL overall	43.4 (30.9)
Medical resources	59.1 (32.2)
ZCMEI-21 score—points (SD)	
Total score	27.5 (13.9)
Ear symptoms	4.3 (3.6)
Hearing	9.9 (4.6)
Psychosocial impact	11.3 (7.0)
Medical resources	2.0 (2.0)
Hearing	
Mean BC—dB (SD)	27.1 (19.7)
Mean AC—dB (SD)	49.6 (24.1)
Mean ABG—dB (SD)	22.5 (12.4)

*AC* air conduction, *BC* bone conduction, *COM* chronic otitis media, *PTA* pure-tone average, *SD* standard deviation

### Response distribution

**Fig. 1:**
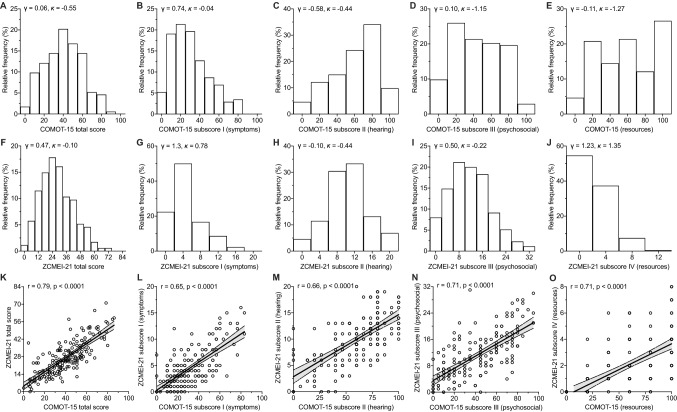
Response distribution and convergent validity of the COMOT-15 and the ZCMEI-21. **a**–**e** Histograms showing the distribution of the COMOT-15 total score (**a**) and subscores (**b**–**e**). Note that **e** (resources) refers to only one question (no. 15). **f–j** Histograms showing the distribution of the ZCMEI-21 total score (**f**) and the subscores (**g**–**j**). γ: skewness, κ: kurtosis. **k**–**o** Convergent validity based on Spearman’s rank correlation between the COMOT-15 and the ZCMEI-21 total score and subscores. Solid line represents linear regression line, dashed line represents 95% prediction interval. *r*: Spearman’s rank correlation coefficient

The COMOT-15 and the ZCMEI-21 generally showed normally distributed scores (Fig. 1a–j) without strong floor and ceiling effects (Table 2) in the total scores and subscores. In particular, the total scores were well distributed with the COMOT-15 having a skewness of 0.06 and a kurtosis of − 0.56 and the ZCMEI-21 having a skewness of 0.47 and a kurtosis of − 0.10 (Fig. 1a/f). Both total scores had a floor and ceiling effect of < 1% (Table 2). Within the subscores, both the symptoms subscores showed a slightly left-skewed distribution (Fig. 1b/g), whereas the hearing subscores showed a slight right skew (Fig. 1c/h). Both the symptoms subscores and the ZCMEI-21 hearing subscore showed a small floor effect of < 10% (Table 2). In contrast, the COMOT-15 hearing subscore showed both a floor effect (5.2%) and a ceiling effect (7.5%). The psychosocial subscores were both slightly left-skewed, with the COMOT-15 subscore plateauing at middle scores (kurtosis: − 1.15), whereas the ZCMEI-21 subscore had a kurtosis closer to a normal distribution (kurtosis: − 0.22). The COMOT-15 medical resource subscore showed a high ceiling effect (26.6%) whereas the ZCMEI-21 medical resource subscore had a high floor effect (23.0%).

**Table 2:** Floor and ceiling effects as well as Cronbach’s α of the COMOT-15 and ZCMEI-21 total score and subscores

		Ear symptoms	Hearing	Psychosocial impact	HRQoL	Medical resources	Total score
COMOT-15	Questions (*n*)	6	3	4	1	1	13
	Floor (*n* [%])	6 (3.5)	7 (4.0)	9 (5.2)	17 (9.8)	8 (4.6)	1 (0.6)
	Ceiling (*n* [%])	0 (0.0)	13 (7.5)	0 (0.0)	0 (0.0)	46 (26.6)	0 (0.0)
	α	0.75	0.94	0.89	na	na	0.89

ZCMEI-21	Questions (*n*)	5	5	8	na	3	21
	Floor (*n* [%])	17 (9.8)	6 (3.4)	6 (3.4)	na	40 (23.0)	1 (0.6)
	Ceiling (*n* [%])	0 (0.0)	1 (0.6)	0 (0.0)	na	0 (0.0)	0 (0.0)
	α	0.71	0.73	0.84	na	0.66	0.88

### Reliability

Cronbach’s α was calculated for every subscore containing more than one question. The results were comparable among the questionnaires and showed satisfactory values ≥ 0.70 and ≤ 0.90 in all but two subscores which implies good internal consistency for both questionnaires (Table 2). As the exceptions, the ZCMEI-21 medical resources subscore had a Cronbach’s α of 0.66. Further, Cronbach’s α of the COMOT-15 hearing subscore was 0.94 indicating a subscore exhibiting unnecessary redundancy.

### Convergent validity

The COMOT-15 and the ZCMEI-21 strongly correlated in the total score (*r* = 0.79, 95% CI 0.72–0.84, *p* < 0.0001, Fig. 1k) as well as in the symptoms subscore (*r* = 0.65, 95% CI 0.55–0.73, *p* < 0.0001, Fig. 1l), the hearing subscore (*r* = 0.66, 95% CI 0.58–0.75, *p* < 0.0001, Fig. 1m), the psychosocial subscore (*r* = 0.71, 95% CI 0.62–0.78, *p* < 0.0001, Fig. 1n) and the medical resources subscore (*r* = 0.71, 95% CI 0.62–0.78, *p* < 0.0001, Fig. 1o). No differences between the two different centers were found for the COMOT-15 total score (mean difference: 4.0, 95%, CI 1.9–9.9, *p* = 0.2) and the ZCMEI-21 total score (mean difference: 2.5, 95%, CI 1.8–6.7, *p* = 0.2).

Using linear regression to model the relationship between the COMOT-15 and ZCMEI-21 total scores, the following equations were found:

$${\mathrm{Total score}}_{\mathrm{ZCMEI}}=0.57 \times {\mathrm{Total score}}_{\mathrm{COMOT}}+4.5.$$

$${\mathrm{Total score}}_{\mathrm{COMOT}}=1.75 \times {\mathrm{Total score}}_{\mathrm{ZCMEI}}-8.1.$$

### Correlation of hearing and HRQoL

**Fig. 2:**
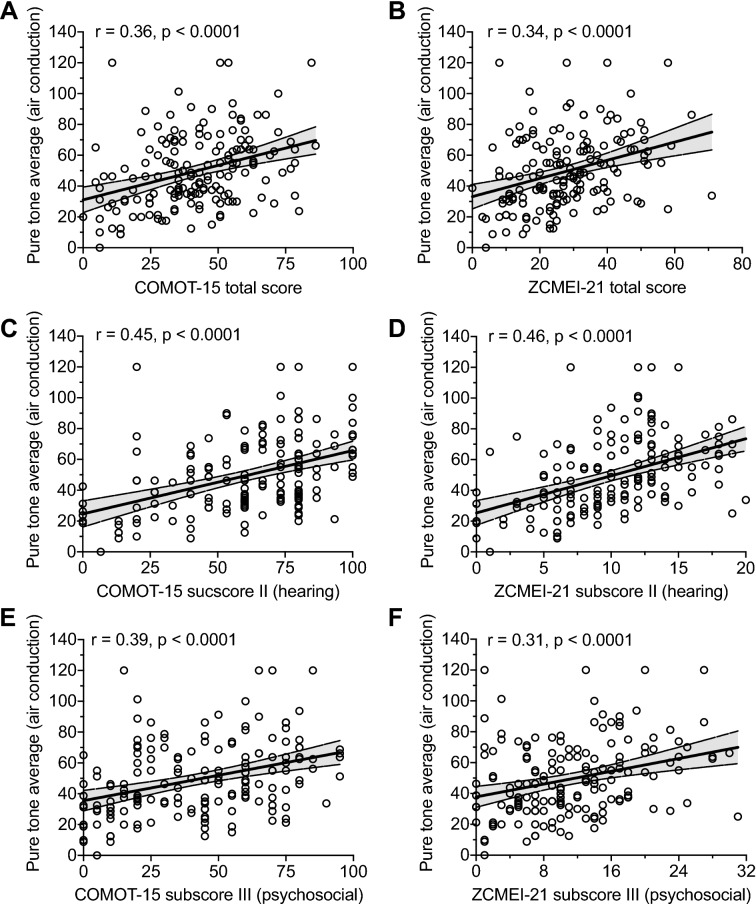
Spearman’s rank correlations between COMOT-15 and ZCMEI-21 scores and hearing. **a**–**b** Correlation between total scores and air conduction pure-tone average. **c**–**d** Correlation between hearing subscores and air conduction pure-tone average. **e**–**f** Correlation between psychosocial subscores and air conduction pure-tone average. Solid line represents linear regression line, dashed line represents 95% prediction interval. *r*: Spearman’s rank correlation coefficient

Fair correlations between the AC PTA and the total scores of both questionnaires (COMOT-15: *r* = 0.36, 95% CI 0.22–0.48, *p* < 0.0001, Fig. 2a; ZCMEI-21: *r* = 0.34, 95% CI 0.19–0.47, *p* < 0.0001, Fig. 2b), the hearing subscores (COMOT-15: *r* = 0.45, 95% CI 0.31–0.56, *p* < 0.0001, Fig. 2c; ZCMEI-21: *r* = 0.46, 95% CI 0.33–0.57, *p* < 0.0001, Fig. 2d) and the psychosocial subscores (COMOT-15: *r* = 0.39, 95% CI 0.25–0.52, *p* < 0.0001, Fig. 2e; ZCMEI-21: *r* = 0.31, 95% CI 0.16–0.45, *p* < 0.0001, Fig. 2f) were found.

### Gender differences

**Fig. 3:**
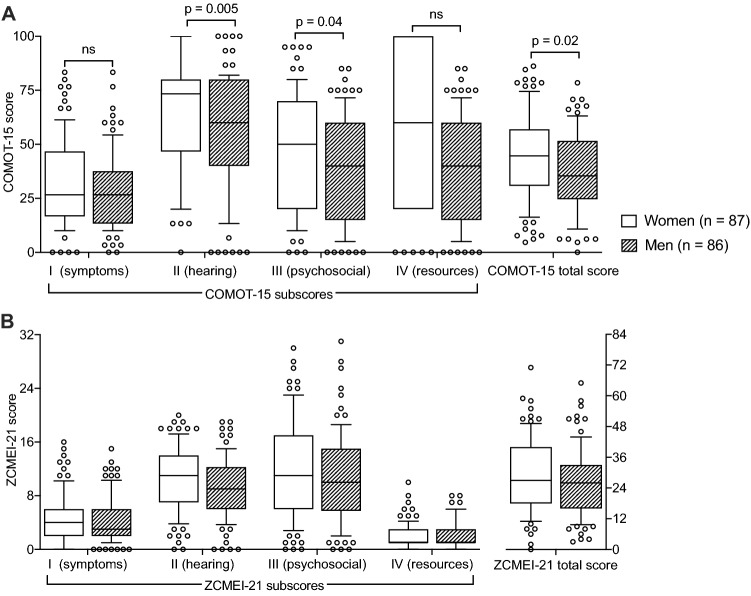
Gender differences. **a** Differences between women and men in the COMOT-15 subscores and total score. **b** Differences between women and men in the ZCMEI-21 subscores and total score. Whiskers indicate 10th–90th percentile

Female scored higher than male patients in the COMOT-15 total score (median difference: 9.2, *p* = 0.02, Fig. 3a), the psychosocial subscore (median difference: 10.0, *p* = 0.04, Fig. 3a), as well as the hearing subscore (median difference: 13.3, *p* = 0.006, Fig. 3a) in contrast to a missing gender difference in the AC threshold (*p* = 0.13). Although similar trends were observed in the ZCMEI-21, no statistically significant differences between men and women were found (Fig. 3b).

## Discussion

The aim of this study was to gather information on the psychometric properties of two instruments assessing HRQoL in COM—i.e. the COMOT-15 and the ZCMEI-21—in a concurrent application. Both questionnaires showed normally distributed scores with generally negligible floor and ceiling effects. Both questionnaires exhibited a satisfactory reliability, a high convergent validity, and a fair correlation with hearing. The COMOT-15 and the ZCMEI-21 differed in revealing gender differences with significant differences between women and men only detected in the COMOT-15 total score and several subscores.

An important psychometric feature of an instrument assessing HRQoL is reliability, i.e. whether the instrument produces consistent results under similar conditions. Cronbach’s α describing the internal consistency was used as an indicator of reliability. Cronbach’s α had acceptable values among the total scores and subscores of both questionnaires, which implies satisfactory internal consistency. These values are also in accordance with their initial evaluations [23, 24]. However, one exception is the COMOT-15 hearing subscore that showed relatively high floor and ceiling effects and a Cronbach’s α > 0.9. This subscore may therefore not reliably distinguish among patients, who perceive their hearing impairment either as low or high. Furthermore, since a Cronbach’s α > 0.9 may indicate redundancy (rather than a higher level of internal consistency) [32], the three questions of the COMOT-15 hearing subscore may capture highly similar aspects of hearing.

Due to the focus of the COMOT-15 psychosocial subscore on the psychosocial impairment due to hearing-related symptoms (but not due to other COM-related symptoms), the respective subscore was expected to strongly correlate with the AC PTA. Further, it was anticipated that it would correlate more strongly with the AC PTA than the respective ZCMEI-21 subscore, which assesses the psychosocial impairment by the entire symptom complex of COM, rather than solely focusing on hearing impairment. In accordance to these assumptions, a trend towards a higher correlation of the COMOT-15 psychosocial subscore and the AC PTA than the respective correlation of the ZCMEI-21 psychosocial subscore was found. This correlation may be weakened by the notion that a majority of patients perceive hearing impairment as the preponderant symptom among the symptom complex of COM. Consequently, hearing predominantly impairs HRQoL. In line, a slightly higher correlation between the COMOT-15 total score and the AC PTA was found compared to the respective correlation of the ZCMEI-21 total score and the AC PTA. As a clinical consequence, the COMOT-15 may be suitable for research settings that focus on hearing impairment or hearing improvement, such as ossicular chain reconstruction or implantable active hearing devices. In contrast, the ZCMEI-21 provides a comprehensive assessment of the COM symptom complex and therefore may also be used in research settings, where ear discharge, vertigo or (postoperative) pain should be covered [15, 25].

The ZCMEI-21 medical resource subscore showed a high floor effect indicating rather few demands of medical resources in the investigated cohort. In contrast, the COMOT-15 medical resources subscore (consisting of one question) showed more ceiling effects. This effect may be explained by the ZCMEI-21 medical resource subscore covering not only the number of consultations of an ENT-specialist, but also the use of local and systemic antibiotics. The COMOT-15 medical resource subscore only consists of a question assessing the number of consultations of an ENT-specialist and may therefore underestimate the use of medical resources of an individual patient. The Cronbach’s α in the ZCMEI-21 medical resource subscore was slightly below a satisfactory level of 0.7, which may be explained by the small number of questions (*n* = 3) of this subscore since Cronbach’s α is substantially influenced by the number of items [33].

Based on the content of the COMOT-15 and ZCMEI-21, a strong correlation between the two instruments in the total scores as well the corresponding subscores was expected, i.e. convergent validity. In accordance, moderate to strong correlations between all subscores and the total scores of the COMOT-15 and the ZCMEI-21 were found. Given these strong correlations, a conversion from one questionnaire to the other may be justified in selected situations, e.g. to compare data among different medical centers or to estimate the corresponding score value of the other questionnaire. Yet, this conversion should be treated with caution and should be preferably used only for interpreting the different questionnaire scores.

Assessing gender differences within the individual questionnaires, women tended to score higher in the total score as well as in all subscores compared to men. It is known, that women tend to score worse in the overall assessment of QoL [34, 35] and that women have higher depression rates than men [36, 37]. Depressive disorders have an impact on the assessment of HRQoL independently from objective symptoms such as the hearing or the extent of the middle ear pathology [38]. Furthermore, differences in stress coping strategies exist between men and women [39]. The present study is among the first studies reporting gender differences in HRQoL in COM [40]. Similar effects have been reported for tinnitus as a related (hearing-associated) symptom [41, 42]. However, the reported gender differences in HRQoL in COM were statistically significant only in the COMOT-15 total score, the COMOT-15 psychosocial subscore and the COMOT-15 hearing subscore. No statistically significant differences were found for the ZCMEI-21.

Although no differences in audiometrically assessed hearing were detected between women and men in this cohort, the largest gender difference was detected in the COMOT-15 hearing subscore. This effect may be due to the COMOT-15 hearing subscore covering only challenging listening situations, while general hearing impairment is incorporated into the COMOT-15 symptoms subscore. Consequently, the gender differences may be explained by the above-mentioned different coping strategies in difficult situations [39, 41]. However, if the statistically different values in the COMOT-15 represent clinically important differences remains unclear, although a difference in the total score of almost 10% of the total score appears to be a large difference.

This study is limited by a cross-sectional design determining data at one time point only. However, the study design is considered suitable to gather information on psychometric properties of the COMOT-15 and the ZCMEI-21. Future studies may prospectively validate the findings of this study. Furthermore, determining the questionnaire scores at different time points will enable to concurrently assess the responsiveness of the two questionnaires, e.g. when measuring therapeutic success in the treatment of COM.

In otology, standardized and validated questionnaires assessing HRQoL enable the analysis of further essential aspects of the current health state and outcomes of surgical therapy. HRQoL as a subjective outcome should be considered as complementing audiometry which is the most important semi-objective outcome in otology. In accordance to previous studies, this work showed that hearing (as measured by pure-tone audiometry) is not the only factor affecting HRQoL [17, 43]. Not only (audiometrically and subjectively perceived) impaired hearing but also symptoms such as tinnitus, ear discharge, vertigo or otalgia may negatively influence psyche and social behavior. These aspects are of considerable importance and are registered by HRQoL questionnaires.

## Conclusion

This is the first study assessing information on the psychometric properties of the COMOT-15 and the ZCMEI-21 in a concurrent application. This study showed that both questionnaires exhibit satisfactory psychometric properties with several subtle differences. The COMOT-15 has a strong focus on hearing. However, its hearing subscore was found to be probably redundant with high floor and ceiling effects. Moreover, the COMOT-15 hearing subscore revealed large gender differences which may finally lead to gender differences in the total score. Consequently, the COMOT-15 may be suited for hearing-focused research questions in COM. When applying the COMOT-15, a gender sensitivity needs to be anticipated. The ZCMEI-21 provides a comprehensive assessment of the COM symptom complex and therefore may also be used in research settings, where ear discharge, vertigo or (postoperative) pain should be covered [15, 25]. Yet, since the COMOT-15 and the ZCMEI-21 show an excellent concurrent validity, the use of either of these questionnaires has to be encouraged. Further, converting one questionnaire score to the other may be justified to estimate corresponding total scores.
